# MUSCLE-SPARING VERSUS STANDARD POSTEROLATERAL THORACOTOMY IN NEONATES WITH ESOPHAGEAL ATRESIA

**DOI:** 10.1590/0102-672020180001e1365

**Published:** 2018-07-02

**Authors:** Shahnam ASKARPOUR, Mehran PEYVASTEH, Amir ASHRAFI, Masoud DEHDASHTIAN, Arash MALEKIAN, Mohammad-Reza ARAMESH

**Affiliations:** 1 Department of Pediatric Surgery, Imam Khomeini Hospital; 2 Department of Neonatalogy, Ahvaz Jundishapur University of Medical Sciences, Ahvaz, Khouzestan, Iran

**Keywords:** Esophageal atresia, Esophagus, Thorax, Esôfago, Tórax, Atresia esofágica, .

## Abstract

*****Background*** ****:**

The muscle-sparing thoracotomy (MST) has not yet been thoroughly studied and assessed in comparison to the traditional thoracotomy method in newborns.

*****Aim*** ****:**

To compare the outcomes of MST and standard posterolateral thoracotomy (PLT) in newborns.

*****Methods*** ****:**

Randomized, controlled, double-blind trial on 40 neonates with esophageal atresia, comparing the time of beginning a surgery until seeing the pleura, the duration of hospitalization in the neonatal intensive care unit, the time in ventilator, the time of returning the shoulder function, the time of returning the Moro reflex, and the mortality between the two techniques.

*****Results*** ****:**

The data showed no differences between the two groups in basic information (weight, height, gender, numbers of prematurity neonates and caesarean). The results on the size of the scar in the MST group was significantly lower than in the PLT group. Also, the time of returning the shoulder function in MST group was earlier than in PLT group. There were no significant differences in the duration since the beginning the surgery to see the pleura, the time of being hospitalized in intensive unit, the time that the infant required ventilator, returning time of the Moro reflex in 1^st^ and 3^rd^ months after the operation, and the mortality rates between MST and PLT groups.

*****Conclusion*** ****:**

It seems that the advantages of using MST over PLT procedure in neonates include the earlier shoulder function recovery and also superior cosmetic results.

## RESUMO

*****Racional*** ****:**

A técnica de toracotomia poupadora de músculo (MST) ainda não foi estudada e avaliada em relação ao método tradicional de toracotomia em recém-nascidos.

*****Objetivo*** ****:**

Comparar os resultados da MST e toracotomia posterolateral padrão (PLT) em recém-nascidos*.*

*****Métodos*** ****:**

Ensaio randomizado, controlado, duplamente cego em 40 neonatos com atresia esofágica, comparando o tempo de início da incisão até ver a pleura, a duração da hospitalização na unidade de terapia intensiva neonatal, o tempo em ventilador, o tempo da volta da função do ombro, tempo de retorno do reflexo Moro e mortalidade entre as duas técnicas.

Os dados não mostraram diferenças entre os dois grupos em informações básicas (peso, altura, gênero, número de neonatos de prematuridade e cesariana). Os resultados sobre o tamanho da cicatriz no grupo MST foram significativamente menores do que no grupo PLT. Além disso, o tempo de retorno da função do ombro no grupo MST foi mais precoce do que no grupo PLT. Não houve diferenças significativas na duração desde o início da operação até a pleura ser vista, o tempo de hospitalização em unidade intensiva, o tempo que a criança necessitou de ventilador, retorno do reflexo Moro nos 1º e 3º meses após a operação, e as taxas de mortalidade entre os grupos.

*****Conclusão*** ****:**

As vantagens de usar o procedimento MST sobre PLT em neonatos incluem a recuperação da função do ombro e também resultados cosméticos superiores.

## INTRODUCTION

Esophageal atresia (EA) is a congenital anomaly that its incidence ranges from one in every 2500-4500 live births^16^. Newborns with it periodically suffer from coughing, cyanosis, and shortness of breath and may be affected by pulmonary aspiration during feeding^12^. Recent improvements in survival of the newborns with EA are indebted to the advances in neonatal intensive cares, anesthesia, and the surgical techniques improvements, intravenous feeding, and antibiotics^23^.

Posterolateral thoracotomy (PLT) is the standard procedure for the most thoracic surgeries; it provides a surgeon an appropriate space for exposing the thoracic organs which easily can be extended in needs^11^ ^,^ ^24^. But, in this procedure, the surgeon has to make an incision in at least one (often the latissimus dorsi muscle) and sometimes in several major chest muscles (such as the serratus anterior, the trapezius, and the rhomboid muscles). This can cause considerable complications such as acute postoperative pain, a decline in lung function and also reduced shoulder girdle performance^1,5 ,6 ,21^. Lower patient age brings greater risk of complications. On the other hand, MST has been proposed due to saving the accessories muscle, as a way to reduce pain, maintaining better lung function and reducing respiratory and other postoperative complications^7^ ^,^ ^22^. However, the MST has not yet been thoroughly studied and assessed in comparison to the traditional thoracotomy in newborns^13^.

The aim of this study was to compare the outcomes of MST and PLT approaches in neonates with EA. 

## METHODS

This study was based on block Random sampling (interventional study) conducted in Imam Khomeini Hospital in Ahwaz, Iran, during 2014-2015. After approval by the Ethics Committee of Ahvaz Jundishapur University of Medical Sciences (Ref. No.: IR.AJUMS.REC.1394.565), informed consent was obtained from all parents’ patients. The necessary explanations on the study procedure were given to the parents. Forty newborns with EA were divided into two groups for MST and standard PLT. None of the patient’s operations was an emergency. They were included into two methods using randomized blocks, and each block contained four patients. Those that needed emergency thoracotomy were excluded. Inclusion criteria were all newborns with EA, and the exclusion parents unsatisfied and cases requiring emergency surgery. 

The evaluated variables were: the time of reaching the pleural cavity by the surgeon, the number of days that the neonatal required ventilator, the days of hospitalization in the intensive care unit, the duration of staying in pediatric surgery section, the infection occurrence in the wound, the size of the surgical scar, the recovery time of shoulder performance, the time of returning to Moro reflex in the 1^st^ and 3^rd^ months and the mortality. The study was considered double-blinded trial, due to the fact that the treatment results were analyzed by different physician aside of the surgical team.

### Statistical analysis

Was performed using SPSS software Statistics for Windows, Version 22.0 (Chicago: SPSS Inc, Chicago, Illinois, USA) and we used the Independent samples t-test, Mann-Whitney, and Chi2 for the data analysis. 

## RESULTS

In this study, 40 newborns (22 boys and 18 girls) with EA were divided into two groups of the PLT and MST. There was no significant difference in the basic Information for the two groups (Table 1). The average birth weight in PLT group was 2577±568 g and it was 2642±594 g in MST group respectively, no significant difference was found between them (p=0.470). Also the mean height in PLT group was 46±4 cm and in MST groups 47±2 cm, without significant difference (p=0.316).

The average time to reach the pleura in PLT technique was 8.6±1.6 min and 7.8±1.5 min in MST group (p=0.229). The remained scar size by PLT was 6.3±0.8 cm and 5.5±1.1 cm in MST group, with no significant difference (p= 0.001). Also, the mean time of returning the shoulder function in newborns in PLT group was 4.7±2.7 days and 2.7± 0.6 days in MST; in this case there was significant difference between the two groups (p=0.001). The average time requiring ventilator in PLT approach was 3.8±1.8 days and in the MST 4.4±1.8 days (p =0.237). Average need to be monitored in the intensive care unit in the PLT group was 11.1±1.9 days and in MST 11.2±1.9 days, without significant difference (p=0.710). The mean duration of hospitalization in the PLT group was16.7±2.6 days and in MST, 17.1±3.0 days (p=0.699). The results of the study revealed that during the hospitalization four patients in each group had surgical-wound-infection. Moro reflex in one-month follow-up was missed only in one case in PLT group, and within three months after discharge, all infants had normal Moro reflex with no significant relationship was found (one-month follow-up p=1.00). No mortality occurred during the operation in the two methods (Table 2).

**TABLE 1:** The infants’ basic info in both groups

P	MST	PLT	
0.470	2642±594	2577±568	Weight (g)
0.316	47±2	46±4	Height (cm)
0.751	(60) 12	(50) 10	Boy (percent)
0.301	(20) 4	(40) 8	Preterm (percent)
1.00	(45) 9	(40) 8	CS Caesarean section (percent)

**TABLE 2:** The comparison of outcomes of MST and PLT approaches

p	MST	PLT	
0.229	7.8±1.5	8.6±1.6	Surgical start time until seeing the pleura (min)
=0.001	5.5±1.1	6.3±0.8	Size (cm)
0.001	2.7±0.6	4.7±2.7	Shoulder function recovery (days)
0.237	4.4±1.8	3.8±1.3	Require a ventilator (days)
0.710	11.2±1.9	11.1±1.9	Intensive care uint (days)
0.699	17.1±3.0	16.7±2.6	Hospitalization (days)
1.00	(20) 4	(20) 4	Surgical wound infection (percent)
1.00	(100) 20	(95) 19	Returning the Moro reflex in the 1st month (percent)
-	(100) 20	(100) 20	Returning the Moro reflex in the third month (percent)
-	0 (0) 0	(0) 0	Mortality

## DISCUSSION

The main therapy for EA is the surgical intervention. But according to age, the absence of full development of other organs, and simultaneous anomalies, the surgical intervention may have known complications^16^. These complications also may occur at the same time in infants without congenital anomalies and immature even with careful surgical technique and proper treatment after surgery^16^. Early complications may occur due to surgical techniques as well as the patient’s specific condition^10^. MST has been developed due to superior cosmetic results after the operation and reducing soft tissue injury, postoperative pain, and complications^16^ ^,^ ^17^. Another advantage of this method that was mentioned in the studies, is keeping the major muscles of the chest wall to minimize complications, such as empyema and bronchopleural fistula^8^ ^,^ ^14^.

In this study, there was no significant difference between the mean time to reach the pleural in MST and PLT in newborns. In similar study on adults, there were different reports in term of time of operations in MST and PLT approaches. Nosotti and colleagues^19^ and Athanassiadi and colleagues^3^ reported that there was no difference in the duration of thoracotomy in the conventional and MST methods while in Akcali and colleagues^1^ and Sugi et al.^25^ the site opening in the patients in PLT approach was significantly less than in MST. The surgeon skills and the available tools can be mentioned as reasons that could cause the difference in this parameter. 

In this study, was found that the size of the scar left by the thoracotomy incision in infants treated with MST is significantly smaller than those treated with PLT. Sugi et, al^25^ reported the size of thoracotomy in MST was also significantly less than in PLT which is in line with this study, while Nosotti and colleagues^19^ found no difference between the incision sizes in the two groups. It seems that the size of the incision depends greatly on the surgeon skills and the type of surgery.

The average time of shoulder function recovery in infants in MST was significantly shorter than in the other group. Akcali et al study^1^ showed that the strength of the latissimus dorsi and the anterior serratus muscles are the same immediately after the surgery in both groups but after one week the muscles strength in MST group against to conventional thoracotomy group is higher and has a significant difference. Also according to Hamilton findings^9^, like in Sugi et al.^25^ the thoracotomy with saving latissimus dorsi and anterior serratus easily returns the arm function, as was found here. 

Our review showed that the need of the patients to intensive care monitoring, as well as the average duration of hospitalization, were not significantly different in the two groups. In Kucukarslan and colleagues^13^ trial studying children with an average age of 4.2 years submitted to thoracotomy, the duration of hospitalization in intensive care unit was less in MST with significant difference. This difference can be considered critical and vital to the infants. Also, in Alavi et al.^2^ paper which is in line with this study they found no significant difference between the two groups in term of the length of hospitalization. In contrary, Miyata et al^15^, Kucukarslan et al ^13^, and Nosotti et al ^19^ reported this duration for MST significantly different and shorter than PLT group. In terms of thoracotomy complications, different studies concluded that there was no significant difference in rates in both groups^19^ ^,^ ^25^ ^,^ ^26^. Infection rate in this study was the same in the two groups. 

## CONCLUSION

The advantages of using MST over PLT procedure in neonates include the earlier shoulder function recovery and, also, superior cosmetic results.
